# Role of Environmental Temperature on the Attack rate and Case fatality rate of Coronavirus Disease 2019 (COVID-19) Pandemic

**DOI:** 10.1080/20008686.2020.1792620

**Published:** 2020-07-16

**Authors:** Mohammad M. Hassan, Mohamed E. El Zowalaty, Shahneaz A. Khan, Ariful Islam, Md. Raihan K. Nayem, Josef D. Järhult

**Affiliations:** a Faculty of Veterinary Medicine, Chattogram Veterinary and Animal Sciences University, Bangladesh; b Zoonosis Science Center, Department of Medical Biochemistry and Microbiology, Uppsala University, Uppsala, Sweden; c Department of Clinical Sciences, College of Medicine, University of Sharjah, Sharjah, UAE; d Centre for Integrative Ecology, School of Life and Environmental Science, Deakin University, Geelong, Australia; e Zoonosis Science Center, Department of Medical Sciences, Uppsala University, Uppsala, Sweden

**Keywords:** SARS-CoV-2, coronaviruses, COVID-19, environmental temperature, pandemic, attack rate, case fatality rate

## Abstract

SARS-CoV-2 is a zoonotic *Betacoronavirus *causing the devastating COVID-19 pandemic. More than twelve million COVID-19 cases and 500 thousand fatalities have been reported in 216 countries. Although SARS-CoV-2 originated in China, comparatively fewer people have been affected in other Asian countries than in Europe and the USA. This study examined the hypothesis that lower temperature may increase the spread of SARS-CoV-2 by comparing attack rate and case fatality rate (until 21 March 2020) to mean temperature in January–February 2020. The attack rate was highest in Luxembourg followed by Italy and Switzerland. There was a significant (p = 0.02) correlation between decreased attack rate and increased environmental temperature. The case fatality rate was highest in Italy followed by Iran and Spain. There was no significant correlation between the case fatality rate and temperature. This study indicates that lower temperature may increase SARS-CoV-2 transmission (measured as an increased attack rate), but there is no evidence that temperature affects the severity of the disease (measured as case fatality rate). However, there are clearly other factors that affect the transmission of SARS-CoV-2, and many of these may be sensitive to interventions, e.g. through increased public awareness and public health response.

## Introduction

Emerging infectious diseases, including infections caused by coronaviruses, continue to pose a threat to public health globally in recent years. Severe acute respiratory syndrome (SARS) and Middle East respiratory syndrome (MERS) are two coronaviral diseases with demonstrated potential to generate significant outbreaks [1]. In particular, MERS continues to pose a significant threat in the Middle East since its emergence in Saudi Arabia in 2012. SARS-CoV-2 is a zoonotic *Betacoronavirus*, which likely originated from bats and is now transmitted between humans. It emerged in late December 2019 in Wuhan in China and causes COVID-19 pandemic which is the first coronavirus pandemic causing global challenges. SARS coronavirus-2 (SARS-CoV-2) emerged in Wuhan, China, in late December 2019. A cluster of patients reported and presented with a common link in a seafood wholesale market (fish market with live animals of different species, including wildlife being sold) in Wuhan (Hubei, China), with pneumonia of initially unknown origin, and the etiologic agent was quickly identified as a novel coronavirus [2,3], which was later designated as SARS-CoV-2 [4]. SARS-CoV-2 causes Coronavirus disease-2019 (COVID-19), an acute respiratory disease, and represents the most recent introduction of a high pathogenic coronavirus into the human population. SARS-CoV-2 is the seventh coronavirus known to infect humans. The emergence of SARS-CoV-2 as a human coronavirus came after the emergence of MERS-CoV in less than a 10-year period. This recent emergence of a previously unknown coronavirus in China has led to huge impacts on humans globally. SARS-CoV-2 and the subsequent COVID-19 pandemic resulted in devastating health and economic impacts on humans [5]. SARS-CoV-2 has caused more than twelve million reported and confirmed cases and more than 500 thousands fatalities in 216 countries around the globe, with more than three million reported cases in the USA as of 9 July 2020 [6,7].

Despite recent efforts in basic and translational influenza and coronavirus research, there is still no vaccine against coronavirus for use in humans including SARS and MERS [1]. COVID-19 now reached the required epidemiological standards for a pandemic [8]. To mitigate an emerging pandemic infectious disease like COVID-19, assessing transmissibility is crucial. The large movement of people in the Asian region and between China and globally due to the lunar new year celebrated in China likely increased the rapid geographical dispersal of infection during the initial outbreak [9]. Relatively low environmental temperature may have increased the spread of the virus in China. Similarly, countries with relatively low environmental temperature may be more affected than high environmental temperature countries during the recent outbreak due to better viral survival. There are few studies available on global risk factors of COVID-19 transmission and pattern of spread [10,11,12,13]. Rigorous testing and case-based interventions have so far formed key pieces of successful control efforts, e.g. in Singapore and Hong Kong [14]. Many other countries are adopting measures termed ‘social distancing’ or ‘physical distancing’, including closing schools and workplaces, limiting gatherings, using face masks, avoiding physical contact when greeting, and implementing cough etiquette. In the initial phase, the infection was epidemic with an estimated basic reproduction number (*R_0_*) of 2.2 and the case number doubled on average every 7.4 days [15]. China was able to reduce the cases more than 90% by implementing strict containment measures. Other affected countries like Italy, Spain, and Iran have not been able to successfully replicate this containment strategy [16]. To further test the hypothesis of temperature affecting the transmission rate, the present study aimed to investigate the correlation of environmental temperature to attack rate and case fatality rate of COVID-19.

## Materials and methods

### Data

We extracted population data (total population in 2019) for the affected countries from the world population review database. We extracted COVID-19 data regarding new cases, total cases, and total deaths from the World Health Organization (WHO) database [17]. We included WHO data from January 2020 (beginning of the outbreak) until 21 March 2020. Furthermore, we extracted weather data (temperature) from the Trading Economics (monthly average temperature data by country). We used the mean temperature of January and February 2020 for each country as a measure of environmental temperature during the study period. There are variations in data recording and disease-tracking systems in different countries, which we consider a limitation of the study.

### Assessment of attack rate and case fatality rate

Attack rate and case fatality rate were calculated using the formulae [18] described below:

$$\text{AR}=\frac{\text{n}\times 100}{\frac{}{}}N$$

Here, AR is the attack rate, *n* is the number of new cases among the population during the period, and *N* is the population at risk at the beginning of the period.

and case fatality rate was calculated using the formula:

$$\text{CFR}=\frac{\text{n}\times 100}{\frac{}{}}N$$

Here, CFR is the case fatality rate, *n* is the number of death due to the particular disease, and *N* is the total number of cases due to the same disease.

The attack rate was multiplied by 1000 to be expressed as cases per 100,000 population, while the case fatality rate was expressed as percent as per the output from the formula.

### Statistical analysis

Pearson’s coefficient was calculated using the ‘PEARSON’ formula in Excel. The *t*-statistic was calculated using the formula:

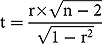

where *t* = *t*-statistic, *r* = absolute value of Pearson’s coefficient, and *n* = number of observations. The *p*-value was calculated from the *t*-statistic in Excel using the ‘TDIST’ function and a two-tailed test.

## Results

**Figure 1.**
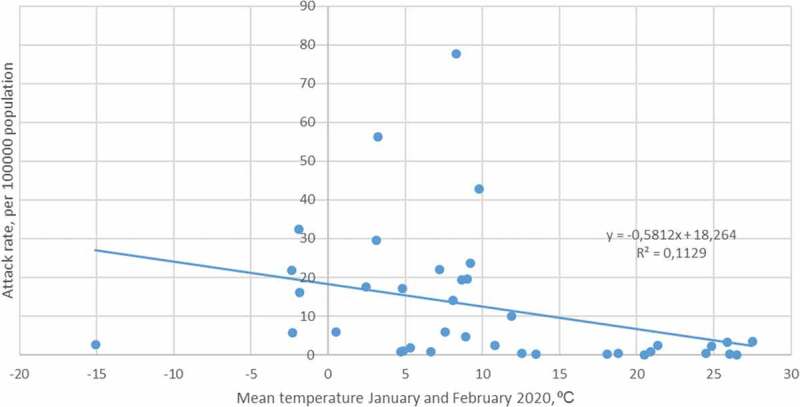
The correlation between environmental temperature and attack rate in different countries. *R* ^2^ = coefficient of determination.

The environmental temperature varied markedly by country based on their geographical location. Some of the Asian countries and most of the European countries and North America experienced low environmental temperatures during the study period. The attack rate (in 100,000 population) of COVID-19 in China was 5.67. However, outside of China, there were countries with markedly higher attack rates: 78.60 in Luxembourg followed by 77.65 in Italy, 56.33 in Switzerland, 42.75 in Spain, 32.38 in Norway, 29.58 in Austria, 23.69 in Iran, 21.93 in Germany, 21.74 in Denmark, 19.55 in Belgium, 19.36 in France, 17.51 in the Netherlands, and 17.17 in South Korea. It was found that the attack rate decreased significantly with increased temperature (*p* = 0.02, *r* = −0.36, *t* = 2.35, DF = 37) (Figure 1).

Mean temperature during January and February 2020, attack rate until March 21, and case fatality rate until March 21 are given in Table 1. The case fatality rate was highest in Italy (8.57%) followed by Indonesia (8.09%), Iran (7.29%), Iraq (7.25%), Spain (5.01%), the UK (4.44%), China (4.00%), France (3.56%), and the Netherlands (3.54%) (Table 1).

**Table 1.** Attack rates and case fatality rates of countries of the continents.

Continent	Countries	Mean Temp (^°^ C) of January and February 2020	Population in 2019	Total cases	Total deaths	AR	CFR
Asia	China	−2.285	1,433,783,686	81,416	3261	5.678	4.006
	India	20.485	1,366,417,754	231	4	0.017	1.732
	Indonesia	26.48	270,625,568	309	25	0.114	8.091
	Iran	9.205	82,913,906	19,644	1433	23.692	7.295
	Iraq	12.565	39,309,783	193	14	0.491	7.254
	Japan	6.67	126,860,301	1007	35	0.794	3.476
	Lebanon	10.815	6,855,713	163	4	2.378	2.454
	Malaysia	25.885	31,949,777	1030	2	3.224	0.194
	Pakistan	13.46	216,565,318	495	3	0.229	0.606
	Philippines	26.025	108,116,615	230	18	0.213	7.826
	South Korea	4.8	51,225,308	8799	103	17.177	1.171
Australia	Australia	27.505	25,203,198	874	7	3.468	0.801
Eurasia	Turkey	4.71	83,429,615	670	9	0.803	1.343
Europe	Austria	3.15	8,955,102	2649	6	29.581	0.227
	Belgium	9.04	390,353	2257	37	19.559	1.639
	Bulgaria	5.31	7,000,119	127	3	1.814	2.362
	Denmark	−2.34	5,771,876	1255	9	21.743	0.717
	France	8.675	65,129,728	12,612	450	19.364	3.568
	Germany	7.245	83,517,045	18,323	45	21.939	0.246
	Greece	8.895	10,473,455	495	8	4.726	1.616
	Ireland	8.11	4,882,495	683	3	13.989	0.439
	Italy	8.295	60,550,075	47,021	4032	77.656	8.575
	Luxembourg	1.25	615,729	484	5	78.606	1.033
	Netherlands	2.45	17,097,130	2994	106	17.512	3.540
	Norway	−1.89	5,378,857	1742	7	32.386	0.402
	Poland	4.895	37,887,768	425	5	1.123	1.176
	Portugal	11.9	10,226,187	1020	6	9.974	0.588
	Spain	9.79	46,736,776	19,980	1002	42.750	5.015
	Sweden	−1.845	10,036,379	1623	16	16.171	0.986
	Switzerland	3.23	8,591,365	4840	43	56.336	0.888
	UK	7.565	67,530,172	3983	177	5.898	4.444
North America	Canada	−15.08	37,411,047	971	12	2.595	1.236
	Costa Rica	24.865	5,047,561	113	2	2.239	1.770
	Mexico	18.095	127,575,529	203	2	0.159	0.985
	USA	0.515	329,064,917	19,624	260	5.964	1.325
South America	Argentina	18.79	44,780,677	158	3	0.353	1.899
	Brazil	24.47	211,049,527	904	11	0.428	1.217
	Ecuador	21.375	17,373,662	426	7	2.452	1.643
	Peru	20.925	32,510,453	263	3	0.809	1.141

Total populations and total cases and total deaths in COVID-19 as of 21 March 2020. The mean temperature of January and February 2020 is included. Only countries with population more than 50,000 and at least 100 total cases with at least two total deaths are included.

AR: attack rate; CFR: case fatality rate.

**Figure 2.**
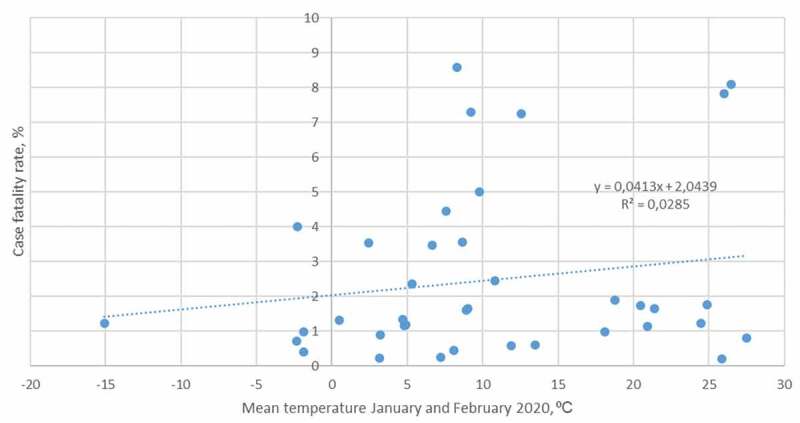
The correlation between environmental temperature and case fatality rate in different countries. *R* ^2^ = coefficient of determination.

The present analysis showed no significant correlation between case fatality rate and environmental temperature (*p* = 0.27, *r* = 0.18, *t* = 1.11, DF = 37) (Figure 2).

## Discussion

SARS-CoV-2 which caused the COVID-19 pandemic originated in China, but interestingly, the neighboring countries with relatively high environmental temperature (Taiwan, Thailand, Myanmar, Nepal, Sri Lanka, and Bangladesh) were relatively less affected than the neighboring countries with low environmental temperature (South Korea, Iran, and Japan). Understanding the stability and transmissibility of viruses in different environmental temperature conditions is critical to recognize the transmission dynamics of a novel infectious agent including SARS-CoV-2 which caused the COVID-19 pandemic. Temperature along with other environmental factors including humidity and ultraviolet radiation is known to affect the transmission of infectious diseases (including viruses) within the community [19,20]. There are also differences in human behavior that likely contribute to the transmission of viruses; for example, spread is more likely in crowded indoor environments [21]. Further, the human immune system may become slightly impaired in cold weather as both vitamin D and melatonin are depleted due to shorter daylight [22].

Early in the COVID-19 outbreak, Asian countries with relatively lower environmental temperature (e.g. South Korea, Japan, Indonesia, and Iran) had more severe outbreaks than warmer countries (e.g. Singapore, Taiwan, Malaysia, Nepal, Bangladesh, and Thailand). In the further spread of COVID-19 also, other countries with low environmental temperature were severely affected in Europe (including Luxembourg, Italy, Spain, Germany, Belgium, the Netherlands, and the UK). Our study demonstrated a significant correlation between environmental temperature and attack rate, where a higher environmental temperature is correlated to a lower COVID-19 attack rate. However, the relatively low *R* ^2^ value indicates that there are also other factors substantially affecting transmission. Temperature is a strong predictor of influenza seasonality in high latitudes, suggesting that cold temperatures may drive seasonal epidemics in these regions [23,24]. Individuals in temperate regions spend the majority of their time indoors where the temperature is managed and does not correlate well with outdoor temperatures. Nevertheless, temperature may affect the timing of influenza epidemics through mechanisms independent of virus survival; for example, low outdoor temperatures may promote indoor crowding, thereby increasing person-to-person contact rates. As the temperature is now increasing in temperate countries in the Northern Hemisphere, and decreasing in the Southern, it will be interesting to follow SARS-CoV-2 transmission over time.

The distance droplets travel depends on the velocity and mechanism by which respiratory droplets are propelled from the source (coughing or sneezing), the density of respiratory secretions, environmental factors such as temperature and humidity, and the ability of the pathogen to maintain infectivity over that distance. The interaction between temperature and humidity on viral activity is challenging to assess [25]. The human coronavirus associated with the common cold was reported to remain viable only for 3 h on environmental surfaces after drying. However, it remains viable for many days in liquid suspension [26]. In aerosolized form, human coronavirus 229E is generally less stable in high humidity [27]. The virus is stable for 3 weeks at room temperature in a wet environment, but it is easily killed by heat at 56°C for 15 min [28]. SARS-CoV-2 may retain its infectivity up to 2 weeks at low temperatures and low humidity environment. This indicates that SARS CoV-2 may be a stable virus transmittable by indirect contact or fomites [29]. Thus, contaminated surfaces may play a significant role in the transmission of infection in the hospital and the community [30].

Importantly, environmental temperature is not a driver of COVID-19 transmission that is possible to influence, and thus, it is necessary for governments to focus on the implementation of public health interventions in a timely manner. Due to the lack of vaccines and specific antiviral drugs against SARS-CoV-2, the implementation of handwashing and other hygiene-related interventions, restrictions of movements in and between countries [13], and avoiding mass gatherings are plausible measures. The implication of such basic strategies to mitigate SARS-CoV-2 spread may also reduce the significant burden on the economy created by the pandemic [14,31].

## Conclusion

In our study, the lower environmental temperature of a country was correlated to a higher attack rate of SARS-CoV-2. This may explain some of the variations of COVID-19 burden seen in different countries. The environmental temperature had no significant correlation to case fatality rate in our study, suggesting that environmental temperature affects transmission rather than disease severity. As the environmental temperature is not possible to affect transmission, other public health measures are imperative to mitigate the COVID-19 pandemic. Public awareness including personal interventions (movement restriction, social and physical distancing, and hygiene) and increased public health response including case detection, isolation, and contact tracing may help reduce the spread of the virus and decrease the fatalities due to this pandemic.

## References

[1] Abdirizak F , Lewis R , Chowell G. Evaluating the potential impact of targeted vaccination strategies against severe acute respiratory syndrome coronavirus (SARS-CoV) and Middle East respiratory syndrome coronavirus (MERS-CoV) outbreaks in the healthcare setting. Theor Biol Med Modell. 2019;16(1):16. 10.1186/s12976-019-0112-6 PMC6778978 31587665

[2] Zhu N , Zhang D , Wang W , et al. A novel coronavirus from patients with pneumonia in China, 2019. N Engl J Med. 2020;382(8):727–6. . 31978945 10.1056/NEJMoa2001017 PMC7092803

[3] Liu W , Zhang Q , Chen J , et al. Detection of Covid-19 in children in early January 2020 in Wuhan, China1. N Engl J Med. 2020;382(14):1370–1371. . 10.1056/NEJMc2003717 PMC7121643 32163697

[4] Gorbalenya A, Baker S, Baric R et al. The species severe acute respiratory syndrome-related coronavirus: classifying 2019-nCoV and naming it SARS-CoV-2. Nat Microbiol. 2020;536–544 (2020). doi: 10.1038/s41564-020-0695-z PMC7095448 32123347

[5] El Zowalaty ME , Young SG , Järhult JD. Environmental impact of the COVID-19 pandemic – a lesson for the future. Taylor & Francis; 2020. Infec Ecol Epidemiol 10 (1):1768023. doi: 10.1080/20008686.2020.1768023. PMC7448928 32922688

[6] Coronavirus COVID-19 Global Cases by Johns Hopkins, The Center for Systems Science and Engineering (CSSE); 2020. [cited 09 July 2020]. Availabe from: https://gisanddata.maps.arcgis.com/apps/opsdashboard/index.html#/bda7594740fd40299423467b48e9ecf6

[7] Dong E , Du H , Gardner L . An interactive web-based dashboard to track COVID-19 in real time. Lancet Infect Dis. 2020;20(5):533–534. 32087114 10.1016/S1473-3099(20)30120-1 PMC7159018

[8] Callaway E . Time to use the p-word? Coronavirus enter dangerous new phase. Nature. 2020;579:12. doi: 10.1038/d41586-020-00551-1 33623145

[9] Yang Z, Gao W, Zhao X, Hao C, Xie X (2020) Spatiotemporal Patterns of Population Mobility and its Determinants in Chinese Cities Based on Travel Big Data. Sustainability 2020; 12 (10):4012; 10.3390/su121040121.

[10] Wu JT , Leung K , Leung GM . Nowcasting and forecasting the potential domestic and international spread of the 2019-nCoV outbreak originating in Wuhan, China: a modelling study. Lancet. 2020;395(10225):689–697. 32014114 10.1016/S0140-6736(20)30260-9 PMC7159271

[11] Bogoch II , Watts A , Thomas-Bachli A , et al. Potential for global spread of a novel coronavirus from China. J Travel Med. 2020;27(2). doi: 10.1093/jtm/taaa011 PMC7074660 31985790

[12] Chinazzi M, Davis JT, Gioannini C, et al. Preliminary assessment of the International Spreading Risk Associated with the 2019 novel Coronavirus (2019-nCoV) outbreak in Wuhan city. Lab. Model. Biol. Soc.–Techn. Syst; 2020. Available from: https://www.mobs-lab.org/uploads/6/7/8/7/6787877/wuhan_novel_coronavirus__6_.pdf (accessed on 5 April 2020).

[13] Haider N , Yavlinsky A , Simons D , et al. Passengers‘ destinations from China: low risk of novel coronavirus (2019-nCoV) transmission into Africa and South America. Epidemiol Infect. 2020;148:e41. 32100667 10.1017/S0950268820000424 PMC7058650

[14] Anderson RM , Heesterbeek H , Klinkenberg D , et al. How will country-based mitigation measures influence the course of the COVID-19 epidemic? Lancet. 2020;395(10228):931–934. 32164834 10.1016/S0140-6736(20)30567-5 PMC7158572

[15] Li Q , Guan X , Wu P , et al. Early transmission dynamics in Wuhan, China, of novel coronavirus–infected pneumonia. N Engl J Med. 2020;382(13):1199–1207. . 10.1056/NEJMoa2001316 PMC7121484 31995857

[16] Remuzzi A , Remuzzi G . COVID-19 and Italy: what next? Lancet. 2020;395(10231):1225–1228. 10.1016/S0140-6736(20)30627-9 PMC7102589 32178769

[17] Ritchie H, Coronavirus source data. Available at https://ourworldindata.org/coronavirus-source-data. Accessed 22 March 2020.

[18] Kanchan T , Kumar N , Unnikrishnan B . Mortality: statistics. In: Payne-James J , Byard RW , editors. Encyclopedia of forensic and legal medicine. 2nd ed. Oxford: Elsevier; 2016. p. 572–577.

[19] Fares A . Factors influencing the seasonal patterns of infectious diseases. Int J Prev Med. 2013;4(2):128–132. 23543865 PMC3604842

[20] Fernstrom A , Goldblatt M . Aerobiology and its role in the transmission of infectious diseases. J Pathog. 2013;2013:493960. doi:10.1155/2013/493960 PMC3556854 23365758

[21] Pinter-Wollman N , Jelić A , Wells NM . The impact of the built environment on health behaviours and disease transmission in social systems. Phil Trans Royal Soc London Series B Biol Sci. 2018;373(1753):20170245. 10.1098/rstb.2017.0245 PMC6030577 29967306

[22] Carrillo-Vico A , Lardone P , Álvarez-Sánchez N , et al. Melatonin: buffering the immune system. Int J Mol Sci. 2013;14(4):8638–8683. . 23609496 10.3390/ijms14048638 PMC3645767

[23] Tamerius JD , Shaman J , Alonso WJ , et al. Correction: environmental predictors of seasonal influenza epidemics across temperate and tropical climates. PLoS Pathog. 2013;9(11):e1003194-e1003194. doi:10.1371/annotation/df689228-603f-4a40-bfbf-a38b13f88147 PMC3591336 23505366

[24] Lowen AC , Steel J , Schultz-Cherry S . Roles of humidity and temperature in shaping influenza seasonality. J Virol. 2014;88(14):7692–7695. 24789791 10.1128/JVI.03544-13 PMC4097773

[25] Yang W , Marr LC . Mechanisms by which ambient humidity may affect viruses in aerosols. Appl Environ Microbiol. 2012;78(19):6781–6788. 22820337 10.1128/AEM.01658-12 PMC3457514

[26] Chan K-H, Peiris JM, Lam S, et al. The effects of temperature and relative humidity on the viability of the SARS coronavirus. Adv Virol. 2011;2011:734690, doi: 10.1155/2011/734690. PMC3265313 22312351

[27] Geller C , Varbanov M , Duval RE . Human coronaviruses: insights into environmental resistance and its influence on the development of new antiseptic strategies. Viruses. 2012;4(11):3044–3068. 23202515 10.3390/v4113044 PMC3509683

[28] Pirtle E , Beran G . Virus survival in the environment. Rev Sci Tech. 1991;10(3):733–748. 1782426 10.20506/rst.10.3.570

[29] Cheng VCC , Lau SKP , Woo PCY , et al. Severe acute respiratory syndrome coronavirus as an agent of emerging and reemerging infection. Clin Microbiol Rev. 2007;20(4):660–694. . 17934078 10.1128/CMR.00023-07 PMC2176051

[30] Otter JA , Yezli S , French GL The role of contaminated surfaces in the transmission of nosocomial pathogens. In: Use of biocidal surfaces for reduction of healthcare acquired infections, edited by Borkow G. Springer, Springer International Publishing, USA; 2014. p. 27–58. Available at: 10.1007/978-3-319-08057-4_3.

[31] Koo JR , Cook AR , Park M , et al. Interventions to mitigate early spread of SARS-CoV-2 in Singapore: a modelling study. Lancet Infect Dis. 2020;20(6):678–688. 10.1016/S1473-3099(20)30162-6 PMC7158571 32213332

